# Tetra-*n*-propyl­ammonium chloride monohydrate

**DOI:** 10.1107/S1600536809009532

**Published:** 2009-03-25

**Authors:** Yun-Xia Yang, Qi Li, Seik Weng Ng

**Affiliations:** a College of Chemistry, Beijing Normal University, Beijing 100875, People’s Republic of China; b Department of Chemistry, University of Malaya, 50603 Kuala Lumpur, Malaysia

## Abstract

The crystal structure of the title salt hydrate, C_12_H_28_N^+^·Cl^−^·H_2_O, consists of non-inter­acting cations and anions. The water mol­ecule forms hydrogen bonds to two chloride ions, about a center of inversion, generating a planar eight-membered {⋯H—O—H⋯Cl}_2_ ring.

## Related literature

For the corresponding undecahydrated fluoride, see: Lipkowski *et al.* (1992 ▶, 1997 ▶). For the anhydrous bromide, see: Zalkin (1957 ▶). For the anhydrous iodide, see: Yoshida *et al.* (1994 ▶)
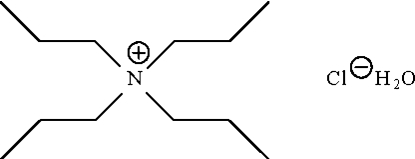


## Experimental

### 

#### Crystal data

C_12_H_28_N^+^·Cl^−^·H_2_O
*M* *_r_* = 239.82
Monoclinic, 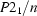

*a* = 8.4228 (2) Å
*b* = 17.4383 (4) Å
*c* = 10.6885 (2) Å
β = 97.892 (1)°
*V* = 1555.05 (6) Å^3^
*Z* = 4
Mo *K*α radiation
μ = 0.23 mm^−1^
*T* = 295 K
0.60 × 0.40 × 0.35 mm

#### Data collection

Bruker SMART APEXII diffractometer
Absorption correction: multi-scan (*SADABS*; Sheldrick, 1996 ▶) *T*_min_ = 0.769, *T*_max_ = 1.000 (expected range = 0.710–0.923)
9762 measured reflections
3562 independent reflections
2719 reflections with *I* > 2σ(*I*)
*R*_int_ = 0.021

#### Refinement

*R*[*F* ^2^ > 2σ(*F* ^2^)] = 0.048
*wR*(*F* ^2^) = 0.150
*S* = 1.02
3562 reflections
144 parameters
3 restraints
H atoms treated by a mixture of independent and constrained refinement
Δρ_max_ = 0.30 e Å^−3^
Δρ_min_ = −0.26 e Å^−3^

### 

Data collection: *APEX2* (Bruker, 2008 ▶); cell refinement: *SAINT* (Bruker, 2008 ▶); data reduction: *SAINT*; program(s) used to solve structure: *SHELXS97* (Sheldrick, 2008 ▶); program(s) used to refine structure: *SHELXL97* (Sheldrick, 2008 ▶); molecular graphics: *X-SEED* (Barbour, 2001 ▶); software used to prepare material for publication: *publCIF* (Westrip, 2009 ▶).

## Supplementary Material

Crystal structure: contains datablocks global, I. DOI: 10.1107/S1600536809009532/tk2397sup1.cif

Structure factors: contains datablocks I. DOI: 10.1107/S1600536809009532/tk2397Isup2.hkl

Additional supplementary materials:  crystallographic information; 3D view; checkCIF report

## Figures and Tables

**Table 1:** Hydrogen-bond geometry (Å, °)

*D*—H⋯*A*	*D*—H	H⋯*A*	*D*⋯*A*	*D*—H⋯*A*
O1—H11⋯Cl1	0.86 (1)	2.37 (1)	3.227 (2)	175 (2)
O1—H12⋯Cl1^i^	0.86 (1)	2.51 (1)	3.352 (2)	168 (3)

Symmetry code: (i) 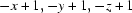.

**Fig. 1.**
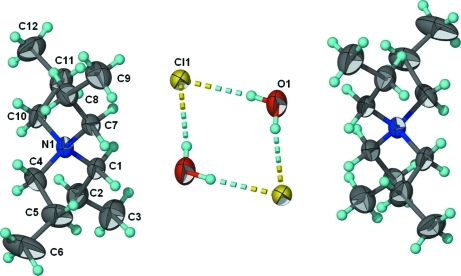
Thermal ellipsoid plot (Barbour, 2001) of the asymmetric unit of the title compound and its centrosymmetric mate; displacement ellipsoids are set at the 50% probability level. Hydrogen atoms are drawn as spheres of arbitrary radius. Dashed lines denote hydrogen bonds.

## supplementary crystallographic information

### Experimental

The salt was one of the possible products of the reaction of tetra-*n*-propylammonium hydroxide, guanidnium chloride and 1,3,5-tri(4-carboxyphenyl)benzene in a water/ethanol mixture. The other products were not identified.

### Refinement

Carbon and nitrogen-bound H-atoms were placed in calculated positions (C—H 0.96–0.97 Å) and were included in the refinement in the riding model approximation, with *U*_iso_(H) = 1.2*U*_eq_(C).

The water H-atoms were located in a difference Fourier map, and were refined with distance restraints of O–H 0.85±0.01 Å and H···H 1.39±0.01 Å; their U_iso_ values were refined.

### Crystal data

**Table:**

C_12_H_28_N^+^·Cl^−^·H_2_O	*F*(000) = 536
*M**_r_* = 239.82	*D*_x_ = 1.024 Mg m^−^^3^
Monoclinic, *P*2_1_/*n*	Mo *K*α radiation, λ = 0.71073 Å
Hall symbol: -P 2yn	Cell parameters from 3424 reflections
*a* = 8.4228 (2) Å	θ = 2.3–27.8°
*b* = 17.4383 (4) Å	µ = 0.23 mm^−^^1^
*c* = 10.6885 (2) Å	*T* = 295 K
β = 97.892 (1)°	Block, colorless
*V* = 1555.05 (6) Å^3^	0.60 × 0.40 × 0.35 mm
*Z* = 4	

### Data collection

**Table:**

Bruker SMART APEXII diffractometer	3562 independent reflections
Radiation source: fine-focus sealed tube	2719 reflections with *I* > 2σ(*I*)
graphite	*R*_int_ = 0.021
φ and ω scans	θ_max_ = 27.5°, θ_min_ = 2.3°
Absorption correction: multi-scan (*SADABS*; Sheldrick, 1996)	*h* = −10→10
*T*_min_ = 0.769, *T*_max_ = 1.000	*k* = −22→15
9762 measured reflections	*l* = −13→13

### Refinement

**Table:**

Refinement on *F*^2^	Primary atom site location: structure-invariant direct methods
Least-squares matrix: full	Secondary atom site location: difference Fourier map
*R*[*F*^2^ > 2σ(*F*^2^)] = 0.048	Hydrogen site location: inferred from neighbouring sites
*wR*(*F*^2^) = 0.150	H atoms treated by a mixture of independent and constrained refinement
*S* = 1.02	*w* = 1/[σ^2^(*F*_o_^2^) + (0.08*P*)^2^ + 0.2641*P*] where *P* = (*F*_o_^2^ + 2*F*_c_^2^)/3
3562 reflections	(Δ/σ)_max_ = 0.001
144 parameters	Δρ_max_ = 0.30 e Å^−^^3^
3 restraints	Δρ_min_ = −0.26 e Å^−^^3^

### Special details

**Table:**

Experimental. A somewhat large crystal was used in the measurements, but this does not seem to have had an adverse efffect on the quality of the diffraction data.
Geometry. All e.s.d.'s (except the e.s.d. in the dihedral angle between two l.s. planes) are estimated using the full covariance matrix. The cell e.s.d.'s are taken into account individually in the estimation of e.s.d.'s in distances, angles and torsion angles; correlations between e.s.d.'s in cell parameters are only used when they are defined by crystal symmetry. An approximate (isotropic) treatment of cell e.s.d.'s is used for estimating e.s.d.'s involving l.s. planes.

### Fractional atomic coordinates and isotropic or equivalent isotropic displacement parameters (Å^2^)

**Table:**

	*x*	*y*	*z*	*U*_iso_*/*U*_eq_	
Cl1	0.49841 (5)	0.65518 (2)	0.49054 (4)	0.06695 (19)	
O1	0.7153 (2)	0.50593 (9)	0.57075 (19)	0.0885 (5)	
H11	0.661 (3)	0.5471 (8)	0.553 (2)	0.116 (9)*	
H12	0.655 (3)	0.4680 (9)	0.543 (3)	0.148 (13)*	
N1	0.00272 (15)	0.67045 (7)	0.32316 (12)	0.0479 (3)	
C1	0.11887 (19)	0.63243 (9)	0.24594 (14)	0.0509 (4)	
H1A	0.2255	0.6522	0.2742	0.061*	
H1B	0.1205	0.5779	0.2638	0.061*	
C2	0.0833 (3)	0.64325 (11)	0.10428 (16)	0.0693 (5)	
H2A	−0.0220	0.6227	0.0738	0.083*	
H2B	0.0831	0.6975	0.0843	0.083*	
C3	0.2080 (3)	0.60277 (16)	0.0399 (2)	0.0971 (8)	
H3A	0.1855	0.6107	−0.0496	0.146*	
H3B	0.2058	0.5489	0.0579	0.146*	
H3C	0.3121	0.6230	0.0705	0.146*	
C4	−0.1683 (2)	0.64589 (12)	0.27918 (17)	0.0646 (5)	
H4A	−0.2367	0.6678	0.3358	0.077*	
H4B	−0.2012	0.6673	0.1959	0.077*	
C5	−0.1959 (3)	0.56022 (15)	0.2729 (2)	0.0900 (7)	
H5A	−0.1734	0.5384	0.3569	0.108*	
H5B	−0.1240	0.5368	0.2204	0.108*	
C6	−0.3680 (3)	0.5438 (2)	0.2183 (3)	0.1355 (14)	
H6A	−0.3851	0.4894	0.2146	0.203*	
H6B	−0.3893	0.5649	0.1347	0.203*	
H6C	−0.4386	0.5667	0.2708	0.203*	
C7	0.0544 (2)	0.64638 (9)	0.45924 (14)	0.0516 (4)	
H7A	0.0347	0.5918	0.4659	0.062*	
H7B	0.1692	0.6542	0.4787	0.062*	
C8	−0.0258 (2)	0.68710 (12)	0.55843 (16)	0.0659 (5)	
H8A	−0.1408	0.6791	0.5424	0.079*	
H8B	−0.0052	0.7418	0.5556	0.079*	
C9	0.0402 (3)	0.65545 (14)	0.68748 (19)	0.0851 (7)	
H9A	−0.0114	0.6804	0.7511	0.128*	
H9B	0.1536	0.6647	0.7036	0.128*	
H9C	0.0203	0.6013	0.6894	0.128*	
C10	0.0095 (2)	0.75695 (9)	0.30981 (17)	0.0585 (4)	
H10A	−0.0243	0.7701	0.2220	0.070*	
H10B	−0.0671	0.7795	0.3590	0.070*	
C11	0.1705 (3)	0.79260 (11)	0.3505 (2)	0.0802 (6)	
H11A	0.2444	0.7768	0.2933	0.096*	
H11B	0.2122	0.7746	0.4345	0.096*	
C12	0.1596 (3)	0.87896 (12)	0.3513 (2)	0.0921 (7)	
H12A	0.2638	0.9002	0.3782	0.138*	
H12B	0.0873	0.8947	0.4085	0.138*	
H12C	0.1207	0.8969	0.2678	0.138*	

### Atomic displacement parameters (Å^2^)

**Table:**

	*U*^11^	*U*^22^	*U*^33^	*U*^12^	*U*^13^	*U*^23^
Cl1	0.0595 (3)	0.0543 (3)	0.0819 (3)	0.00883 (18)	−0.0084 (2)	−0.0134 (2)
O1	0.0763 (10)	0.0649 (10)	0.1177 (13)	0.0160 (8)	−0.0101 (9)	0.0075 (9)
N1	0.0450 (7)	0.0476 (7)	0.0500 (7)	0.0069 (5)	0.0023 (5)	0.0061 (5)
C1	0.0534 (8)	0.0449 (8)	0.0543 (8)	0.0083 (7)	0.0067 (7)	0.0053 (6)
C2	0.0840 (13)	0.0695 (12)	0.0544 (9)	0.0188 (10)	0.0100 (9)	0.0099 (8)
C3	0.125 (2)	0.1048 (18)	0.0659 (12)	0.0372 (15)	0.0271 (13)	0.0045 (12)
C4	0.0482 (9)	0.0874 (13)	0.0561 (9)	−0.0014 (8)	−0.0001 (7)	−0.0002 (8)
C5	0.0818 (14)	0.0948 (17)	0.0936 (15)	−0.0340 (12)	0.0128 (12)	−0.0136 (12)
C6	0.099 (2)	0.207 (4)	0.103 (2)	−0.078 (2)	0.0222 (16)	−0.055 (2)
C7	0.0525 (8)	0.0511 (9)	0.0495 (8)	0.0052 (7)	0.0011 (6)	0.0064 (6)
C8	0.0613 (10)	0.0782 (12)	0.0572 (10)	0.0087 (9)	0.0040 (8)	−0.0026 (9)
C9	0.0846 (15)	0.1143 (19)	0.0561 (10)	0.0104 (12)	0.0080 (10)	0.0030 (11)
C10	0.0639 (10)	0.0469 (9)	0.0645 (9)	0.0174 (7)	0.0082 (8)	0.0082 (7)
C11	0.0746 (13)	0.0472 (10)	0.1188 (17)	0.0000 (9)	0.0133 (12)	0.0074 (10)
C12	0.133 (2)	0.0500 (11)	0.0923 (15)	0.0012 (12)	0.0120 (14)	−0.0009 (10)

### Geometric parameters (Å, °)

**Table:**

O1—H11	0.858 (10)	C6—H6A	0.9600
O1—H12	0.859 (10)	C6—H6B	0.9600
N1—C4	1.514 (2)	C6—H6C	0.9600
N1—C1	1.517 (2)	C7—C8	1.511 (2)
N1—C10	1.517 (2)	C7—H7A	0.9700
N1—C7	1.5189 (18)	C7—H7B	0.9700
C1—C2	1.514 (2)	C8—C9	1.519 (3)
C1—H1A	0.9700	C8—H8A	0.9700
C1—H1B	0.9700	C8—H8B	0.9700
C2—C3	1.508 (3)	C9—H9A	0.9600
C2—H2A	0.9700	C9—H9B	0.9600
C2—H2B	0.9700	C9—H9C	0.9600
C3—H3A	0.9600	C10—C11	1.501 (3)
C3—H3B	0.9600	C10—H10A	0.9700
C3—H3C	0.9600	C10—H10B	0.9700
C4—C5	1.512 (3)	C11—C12	1.509 (3)
C4—H4A	0.9700	C11—H11A	0.9700
C4—H4B	0.9700	C11—H11B	0.9700
C5—C6	1.514 (3)	C12—H12A	0.9600
C5—H5A	0.9700	C12—H12B	0.9600
C5—H5B	0.9700	C12—H12C	0.9600
			
H11—O1—H12	107.3 (15)	C5—C6—H6C	109.5
C4—N1—C1	111.37 (13)	H6A—C6—H6C	109.5
C4—N1—C10	107.36 (12)	H6B—C6—H6C	109.5
C1—N1—C10	110.37 (12)	C8—C7—N1	116.43 (13)
C4—N1—C7	110.76 (12)	C8—C7—H7A	108.2
C1—N1—C7	106.19 (11)	N1—C7—H7A	108.2
C10—N1—C7	110.82 (12)	C8—C7—H7B	108.2
C2—C1—N1	115.83 (13)	N1—C7—H7B	108.2
C2—C1—H1A	108.3	H7A—C7—H7B	107.3
N1—C1—H1A	108.3	C7—C8—C9	108.86 (16)
C2—C1—H1B	108.3	C7—C8—H8A	109.9
N1—C1—H1B	108.3	C9—C8—H8A	109.9
H1A—C1—H1B	107.4	C7—C8—H8B	109.9
C3—C2—C1	110.06 (15)	C9—C8—H8B	109.9
C3—C2—H2A	109.6	H8A—C8—H8B	108.3
C1—C2—H2A	109.6	C8—C9—H9A	109.5
C3—C2—H2B	109.6	C8—C9—H9B	109.5
C1—C2—H2B	109.6	H9A—C9—H9B	109.5
H2A—C2—H2B	108.2	C8—C9—H9C	109.5
C2—C3—H3A	109.5	H9A—C9—H9C	109.5
C2—C3—H3B	109.5	H9B—C9—H9C	109.5
H3A—C3—H3B	109.5	C11—C10—N1	115.39 (13)
C2—C3—H3C	109.5	C11—C10—H10A	108.4
H3A—C3—H3C	109.5	N1—C10—H10A	108.4
H3B—C3—H3C	109.5	C11—C10—H10B	108.4
C5—C4—N1	115.29 (16)	N1—C10—H10B	108.4
C5—C4—H4A	108.5	H10A—C10—H10B	107.5
N1—C4—H4A	108.5	C10—C11—C12	111.21 (18)
C5—C4—H4B	108.5	C10—C11—H11A	109.4
N1—C4—H4B	108.5	C12—C11—H11A	109.4
H4A—C4—H4B	107.5	C10—C11—H11B	109.4
C4—C5—C6	109.7 (2)	C12—C11—H11B	109.4
C4—C5—H5A	109.7	H11A—C11—H11B	108.0
C6—C5—H5A	109.7	C11—C12—H12A	109.5
C4—C5—H5B	109.7	C11—C12—H12B	109.5
C6—C5—H5B	109.7	H12A—C12—H12B	109.5
H5A—C5—H5B	108.2	C11—C12—H12C	109.5
C5—C6—H6A	109.5	H12A—C12—H12C	109.5
C5—C6—H6B	109.5	H12B—C12—H12C	109.5
H6A—C6—H6B	109.5		
			
C4—N1—C1—C2	54.78 (19)	C4—N1—C7—C8	−68.64 (19)
C10—N1—C1—C2	−64.37 (18)	C1—N1—C7—C8	170.30 (15)
C7—N1—C1—C2	175.45 (15)	C10—N1—C7—C8	50.42 (19)
N1—C1—C2—C3	179.85 (18)	N1—C7—C8—C9	179.81 (16)
C1—N1—C4—C5	53.15 (19)	C4—N1—C10—C11	179.35 (16)
C10—N1—C4—C5	174.08 (16)	C1—N1—C10—C11	−59.08 (19)
C7—N1—C4—C5	−64.80 (19)	C7—N1—C10—C11	58.3 (2)
N1—C4—C5—C6	−175.66 (17)	N1—C10—C11—C12	−171.82 (17)

### Hydrogen-bond geometry (Å, °)

**Table:**

*D*—H···*A*	*D*—H	H···*A*	*D*···*A*	*D*—H···*A*
O1—H11···Cl1	0.86 (1)	2.37 (1)	3.227 (2)	175 (2)
O1—H12···Cl1^i^	0.86 (1)	2.51 (1)	3.352 (2)	168 (3)

Symmetry codes: (i) −*x*+1, −*y*+1, −*z*+1.
